# Understanding long COVID: prevalence, characteristics, and risk factors in the Eastern Province of Saudi Arabia

**DOI:** 10.3389/fmed.2024.1459583

**Published:** 2024-10-04

**Authors:** Adam F. Aldhawyan, Mohammed A. BuSaad, Nawaf E. Almaghlouth, Abdullah H. Alnasser, Jomana A. Alnasser, Abdulelah H. Almansour, Khalid S. AlHarkan

**Affiliations:** ^1^Department of Family and Community Medicine, College of Medicine, Imam Abdulrahman Bin Faisal University, Dammam, Saudi Arabia; ^2^College of Medicine, Imam Abdulrahman Bin Faisal University, Dammam, Saudi Arabia

**Keywords:** long COVID, post-COVID-19 syndrome, post-COVID condition, ongoing symptomatic COVID-19, COVID-19

## Abstract

**Background:**

The COVID-19 pandemic has significantly raised public health concerns and efforts to limit its spread, impacting societies and health systems worldwide. As challenges persist, the emergence of Long COVID (LC) marks a turning point in understanding the pandemic’s long-term effects.

**Aim:**

This study aimed to determine the prevalence of LC in the Eastern Province of the Kingdom of Saudi Arabia (KSA) and explore factors contributing to its persistence.

**Methods:**

This descriptive, cross-sectional, questionnaire-based study was carried out between December 1, 2023, and March 1, 2024, involving 1,355 patients who recovered from COVID-19. Participants were conveniently chosen and information was gathered through in-person interviews in public settings after obtaining consent.

**Results:**

A majority of the patients (*N* = 1,355; 47.5% female; 93.8% Saudis; mean Age ± SD 33.13 ± 12.60 years) had received three COVID-19 vaccine doses (89.5%). Women experienced 17.4% more LC symptoms than men (*p* < 0.001). The risk of having a higher symptom count increased by 42.5% 12 months after acute COVID-19 infection compared with baseline (<3 months, *p* < 0.001). A higher body mass index (BMI) was associated with more symptoms (1.1% increase per unit, *p* = 0.004). More acute-phase symptoms correlated with more LC symptoms (*p* < 0.001). Higher educational attainment reduced LC risk by 33% (*p* < 0.001). Finally, age and vaccination status had no effect on LC symptoms count (*p* > 0.05).

**Conclusion:**

Sociodemographic and clinical factors contribute differently to the chances of having LC and the count of symptoms. Awareness of such factors could provide insight into improving management, leading to better health outcomes.

## Introduction

Amidst the global upheaval triggered by the coronavirus disease (COVID-19), an extremely contagious respiratory disease caused by the severe acute respiratory syndrome coronavirus 2 (SARS-CoV-2) virus identified first in Wuhan, China in December 2019, the virus rapidly spread worldwide, prompting the World Health Organization (WHO) declared it a worldwide pandemic on March 11, 2020 (1). The toll has been staggering, with over 704 million cases and 7 million deaths recorded worldwide (2). In the Kingdom of Saudi Arabia (KSA) alone, over 841 thousand confirmed cases and 9,640 deaths were attributed to the virus (2). This rapid escalation raised public health concerns and sparked efforts to limit its spread. The COVID-19 pandemic has profoundly affected societies worldwide, exposing and exacerbating social issues such as income inequality, health disparities, and the strain on public health systems. It has also severely disrupted mental health, education, and social interactions. Moreover, the pandemic highlighted significant inequities in access to healthcare, reshaping perceptions of health, resilience, and societal vulnerabilities (3, 4). In investigating COVID-19, researchers have identified varying degrees of illness severity. The majority of individuals experience mild to moderate illness and recover without the need for hospitalization. For instance, a 2022 cohort study involving 162 COVID-19 patients reported that 22.9% were asymptomatic, 74.6% experienced mild to moderate symptoms that did not require hospitalization, and only 2.5% of patients required hospital care (5). The WHO has outlined COVID-19 symptoms, ranging from common signs such as fever, cough, and loss of taste/smell to less frequent symptoms such as sore throat, headache, and skin rash, and serious symptoms such as chest pain and difficulty in breathing (6).

As the world struggles with the ongoing challenges posed by the virus, the advent of Long COVID (LC) marks a significant chapter in our understanding of the enduring impact of this pandemic. The Centers for Disease Control and Prevention (CDC) and the National Institute for Health and Care Excellence (NICE) have undertaken a dynamic process of refining guidelines, defining LC/post-COVID-19 syndrome (PCS) as signs and symptoms develop during or after COVID-19 infection, persist for 12 weeks or more, and cannot be explained by an alternate diagnosis, while ongoing symptomatic COVID-19 as signs and symptoms persist for less than 12 weeks after the initial infection (7, 8). Despite rigorous investigations of the factors contributing to the persistent development of post-COVID-19 complications in some patients, the precise pathophysiological mechanisms underlying LC remain unclear (9, 10). Some leading hypotheses include autoimmunity, immune dysregulation, microembolization, and endothelial activation or dysfunction (9, 10).

Although LC presentations vary, common symptoms include fatigue, respiratory symptoms, hair loss, muscle and joint pain, attention deficits, and headache (11, 12). However, more serious symptoms include renal failure, pulmonary fibrosis, myocarditis, arrhythmia, and more (12). A detailed list of the most common LC symptoms by affected body system can be found in Supplementary Table S1. This broad spectrum of symptoms contributes to variations in the reported prevalence across global populations. A UK study published in February 2023 estimated that approximately 2 million individuals reported experiencing LC symptoms (13). Continuous analyses by the CDC in the US found that during March–April 2024, approximately 18% of adults had persistent COVID-19 symptoms beyond acute presentation (14). A large observational meta-analysis of 1.2 million people reported that 6.2% of patients with symptomatic COVID-19 had LC, which included ongoing respiratory problems (3.7%), persistent fatigue with bodily pain or mood swings (3.2%), and cognitive issues (2.2%) (15). Additionally, among 21,797 patients surveyed in China, 8.89% self-reported experiencing LC symptoms, with 2.92% reporting two or more symptoms. The most commonly reported symptom was Fatigue (3.38%), followed by sleep difficulties (2.20%), hair loss (2.06%), cough (1.74%), and sore throat (1.27%) (11).

Moreover, a meta-analysis and comprehensive review with a sample size of 1,680,003 patients published in November 2022 found that the pooled worldwide prevalence of LC was 0.43. Estimates were 0.54 for hospitalized patients and 0.34 for non-hospitalized individuals (16). The United States of America (0.31%), Europe (0.44%), and Asia (0.51%) were the other regions with high prevalence (16). Additionally, in January 2023, cross-sectional research including 520 Arabic patients residing in the KSA was published; 25% of them had LC and the most common recorded symptoms were cough, anosmia, fatigue, headache, muscle pain, arthritis, and shortness of breath (32, 32, 28, 19, 19, 18, and 17% of LC patients, respectively) (17). However, data from 504 patients at King Abdulaziz University Hospital in Jeddah revealed a 45% frequency of LC (18).

In light of this, LC presents a significant challenge to patients’ wellbeing, inducing long-lasting physical discomfort, cognitive decline, and emotional stress, ultimately reshaping their quality of life, increasing healthcare utilization, and increasing chronic sickness-related unemployment (19, 20). Several risk variables have also been found to increase the probability of developing LC. These factors include demographic risks, comorbidities, age, and severity of the acute COVID-19 infection, and other factors (11, 21).

Throughout this article, we endeavor to shed light on the LC prevalence in the Eastern Provinces of the KSA and explore the factors contributing to its persistence, including demographic variables, comorbidities, and the severity of the initial infection.

## Materials and methods

### Study design

This was a descriptive, cross-sectional, questionnaire-based study conducted from December 1, 2023, to March 1, 2024 among 1,350 COVID-19 recovered patients who are currently residing in the Eastern Province of the KSA, who were conveniently selected and whose information was obtained through face-to-face interviews in public community settings.

### Study sample

This study included all COVID-19 diagnosed patients who were at least 18 years old. Patients who refused to provide consent to participate or all requested information were excluded. The Epi Info software (version 7.0) was used to calculate the sample size for a target population of 162,176 patients who recovered from COVID-19, and an expected frequency of 50% for LC. Given a 5% margin of error and a 95% confidence level, 251 participants were the minimum calculated sample size.

### Data collection

Participants’ responses were collected by trained volunteers who administered the surveys using tablet devices. The 25-question survey was structured using questionnaires from previously published literature (16, 22–24). Family physicians reviewed the wording of the survey to ensure accuracy. Moreover, the questionnaire included questions regarding sociodemographic data such as age, sex, and occupation; medical history related to COVID-19 infection, including a history of medical illness, hospital, or intensive care unit admission; history of smoking; and lastly, questions about COVID-19 lingering manifestations and questions about the LC. The survey model is provided in Supplementary Document S1 for reference.

Acute COVID-19 was defined as the signs and symptoms of COVID-19 that lasted for up to 4 weeks after the acute infection. The LC/PCS is defined as signs and symptoms that develop during or after a COVID-19 infection, persist for at least 12 weeks, and cannot be explained by an alternative diagnosis, while ongoing symptomatic COVID-19 is defined as signs and symptoms that persist for less than 12 weeks after the initial infection (7, 8).

### Statistical analysis

The mean and standard deviation were used to describe continuous variables. The Kolmogorov–Smirnov test of statistical normality was used to assess the statistical normality assumption for the metric variables. The metric variables with statistical Normality assumption violations such as skewness were described using median and interquartile range (IQR) scores. Moreover, the categorically measured variables were described with frequencies and percentages, and multiple response dichotomies analysis was used to describe the variables measured with more than one option, such as COVID-19 symptoms. Generalized estimating equation gamma regression analysis was applied to the reported number (i.e., count) of LC symptoms across time. The data had to be restructured into longitudinal data to account for the effects of time on the GEE analysis. The association between the independent predictor variables in the multivariate analysis and the analyzed outcome variables was expressed as exponentiated beta coefficients (Risk Rates) with their associated 95% confidence intervals. The commercially available SPSS IBM statistical analysis program (version 21) was used for statistical data analysis. The statistical significance level was set at *p* < 0.05.

### Ethical considerations

All participants were informed of their enrolment in the study and participant’s informed written consent was obtained before participation. The Declaration of Helsinki’s ethical standards were followed during data collection, handling, and storage, and all precautions were taken to ensure participant confidentiality. The Institutional Review Board (IRB) of Imam Abdulrahman Bin Faisal University in the Eastern Province of Saudi Arabia gave their approval to the study protocol (IRB Number: IRB-2023-01-320).

## Results

### Sociodemographic characteristics

One thousand three hundred and fifty-five people residing in the KSA were participated, and interview-based questionnaires were completed by those who consented to participate in the study.

Most of the participants (93.8%) were Saudi citizens, and 6.2% were expatriates living and working within the Kingdom; 47.5% were women and the remainder (52.5%) were men. The mean ± SD age for the sample was 33.13 ± 12.60 years. The mean body mass index (BMI) score was measured at 26.34 ± 5.19%. Participants were also asked to indicate their smoking habit status; the findings showed that 7.9% were ex-smokers and 32.5% were current smokers, while most of the sample (59.6%) were never smokers. Finally, 48.3% of the participants reported having two or more comorbidities (Table 1).

**Table 1:** Baseline sociodemographic characteristics of study participants (*n* = 1,355).

Characteristic	Mean (SD) or n(%)
Sex	
Female	643 (47.5)
Male	712 (52.5)
Age (years), mean (SD)	33.13 (12.60)
Age group	
20–30 years	752 (55.5)
31–40 years	279 (20.6)
41–50 years	165 (12.2)
51–60 years	101 (7.5)
≥61 years	58 (4.3)
Body mass index (BMI) level	
Body mass index (BMI), mean (SD)	26.34 (5.19)
Underweight	118 (8.7)
Normal	457 (33.7)
Overweight	513 (37.9)
Obese class I	187 (13.8)
Obese class II	57 (4.2)
Obese class III	23 (1.7)
Marital state	
Never married	645 (47.6)
Ever married	710 (52.4)
Level of education	
High school or less education	475 (35.1)
Diploma degree	99 (7.3)
University degree	729 (53.8)
Higher studies	52 (3.8)
Socioeconomic state level	
Very low	10 (0.7)
Low	88 (6.5)
Medium	795 (58.7)
High	238 (17.6)
Very high	224 (16.5)
Smoking	
Never smoker	807 (59.6)
Former smoker	107 (7.9)
Current smoker	441 (32.5)
Comorbidity (2 or more)	
No	700 (51.7)
Yes	655 (48.3)

SD, standard deviation.

### Prevalence of comorbidities

**Figure 1:**
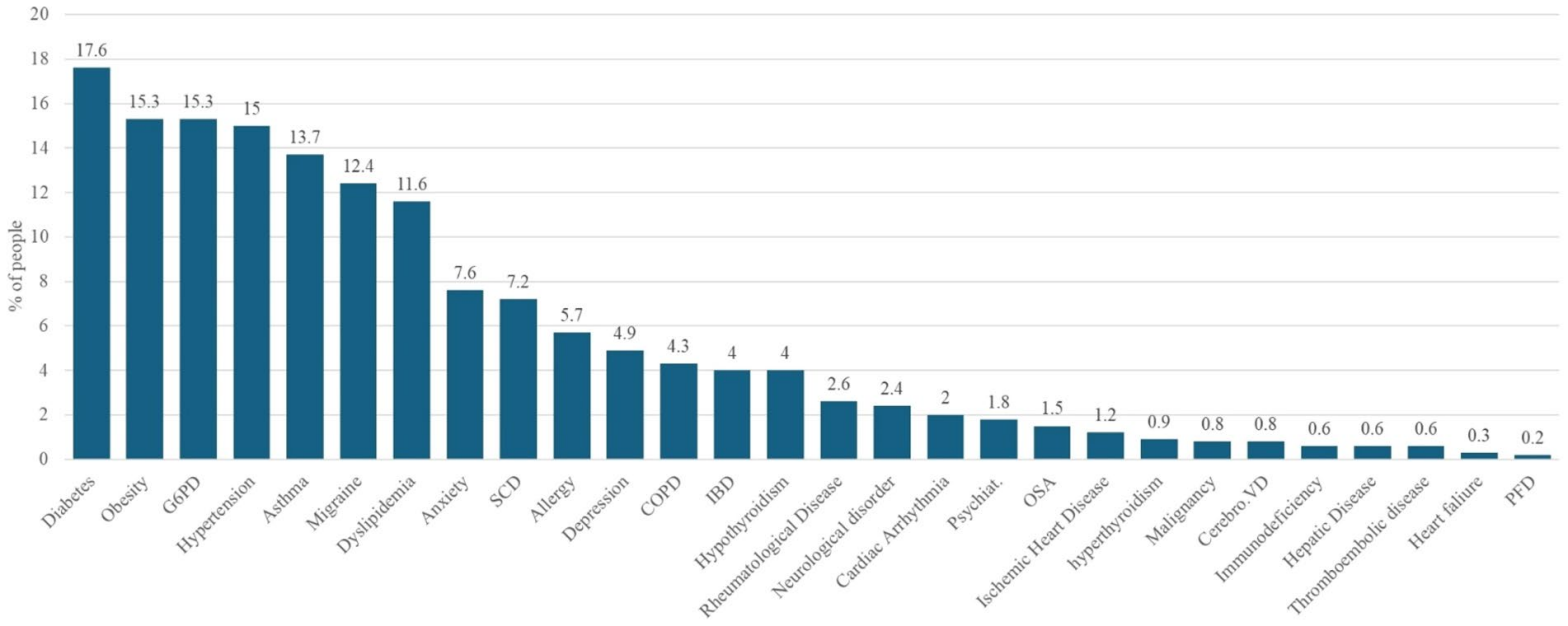
Baseline prevalence of comorbidities among study participants (*n* = 1,355).

Figure 1 shows the prevalence of comorbidities among patients with COVID-19. A notable proportion of the patients exhibited coexisting medical conditions. The most prevalent comorbidities exceeding the 10% threshold were diabetes (17.6%), obesity (15.3%), G6PD deficiency (15.3%), hypertension (15.0%), and asthma (13.7%). These conditions were followed by migraine, which was reported in 12.4% of patients, and dyslipidemia, which affected 11.6% of the study cohort.

### Acute COVID-19 manifestations and clinical characteristics

The patients were asked to state the number of PCR-confirmed COVID-19 infections they had experienced, and the findings showed that 75.9% of them had at least one PCR-confirmed infection. Moreover, the results revealed that the majority (89.5%) had received three COVID-19 vaccine doses. Upon presentation to the hospital, 11.4% of patients had positive evidence of pneumonia. Regarding the need for healthcare, 19% of the patients had no need for any healthcare services, whereas 60.8% needed some form of healthcare that could be managed at home. On the other hand, about 15% of our patients required Emergency Room/Outpatient services, and the remainder, 5.2% of the patients, needed hospital admission (Table 2).

**Table 2:** Acute COVID-19 manifestations and clinical characteristics of study participants (*n* = 1,355).

Characteristic	Median (IQR) or n(%)
How many times did you get PCR confirmed COVID-19 infection?	
Once	1,029 (75.9)
Twice	264 (19.5)
Three times	53 (3.9)
Four times	4 (0.3)
Five times	5 (0.4)
How many COVID-19 vaccine shots did you receive?	
None	9 (0.7)
One dose	10 (0.7)
Two doses	104 (7.7)
Three doses	1,213 (89.5)
Four doses	19 (1.4)
Did you present to the hospital with pneumonia?	
No	1,201 (88.6)
Yes	154 (11.4)
Need for healthcare	
Not needed	258 (19)
Home management	823 (60.7)
ER/Out-patient services	203 (15)
Required hospital admission	71 (5.2)
Type of hospital admission	
Hospital floor admission	50 (3.7)
ICU admission	21 (1.5)
Number of acute COVID-19 symptoms	
Number of acute COVID-19 symptoms, median (IQR)	6 (8)
None/very mild	105 (7.7)
1–3 symptoms	239 (17.6)
4–6 symptoms	350 (25.8)
7–9 symptoms	249 (18.4)
10–15 symptoms	270 (19.9)
≥16 symptoms	142 (10.5)

IQR, interquartile range; PCR, polymerase chain reaction.

### Prevalence of COVID-19 symptoms across acute, ongoing, and LC/PCS phases

Table 3 displays a head-to-head description of the most prevalent COVID-19 symptoms during the acute phase (<1 month) versus the ongoing symptomatic COVID-19 phase (1–3 months) and the LC/PCS phase (≥3 months). Comparing the prevalence of symptoms across the different phases, fever dropped significantly from 74.2% in the acute phase to 2.07% in the LC/PCS phase. Similarly, dyspnea, anosmia, and headache significantly decreased but remained among the most reported symptoms in the LC/PCS phase. Cough, which was reported by 74.2% of patients in the acute phase, witnessed a significant drop in the ongoing symptomatic COVID-19 phase (7%), while still being the most reported symptom. However, in the LC/PCS phase, cough increased in prevalence and was the second most reported symptom (14.24%). Finally, fatigue, which was not the most prevalent symptom in the acute phase, was among the top reported symptoms in the ongoing symptomatic COVID-19 phase (4.1%), and then spiked to rank first in the LC/PCS phase (17.49%).

**Table 3:** Prevalence of COVID-19 symptoms across acute, ongoing, and LC/PCS phases among study participants (*n* = 1,355).

	Acute phase (<1 month)	Ongoing phase (1–3 months)	LC/PCS phase (≥3 months)
Symptom	n (%)	n (%)	n (%)
Cough	928 (74.2)	95 (7)	193 (14.24)
Expectoration	223 (17.8)	11 (0.8)	22 (1.62)
SOB	496 (39.7)	33 (2.4)	78 (5.76)
Dyspnea	331 (47.2)	25 (1.8)	89 (6.57)
Chest pain	324 (25.9)	15 (1.1)	41 (3.03)
Nasal congestion	389 (31.1)	13 (1)	29 (2.14)
Sinusitis	200 (16)	12 (0.9)	44 (3.25)
Fever	953 (76.2)	12 (0.9)	28 (2.07)
Back pain	309 (27.7)	17 (1.3)	63 (4.65)
Joint pain	337 (27)	14 (1)	69 (5.09)
Hypoxia	99 (7.9)	6 (0.4)	14 (1.03)
Ageusia	382 (30.6)	0	58 (4.3)
Dysgeusia	159 (12.7)	0	53 (3.91)
Anosmia	512 (41)	0	131 (9.67)
Hearing problems	28 (2.2)	6 (0.4)	19 (1.4)
Visual problems	21 (1.7)	0	8 (0.59)
Headache	562 (45)	45 (3.3)	102 (7.53)
sleep disturbance	214 (17.1)	14 (1)	56 (4.13)
Excessive sleepiness	144 (11.5)	3 (0.2)	27 (1.99)
Dizziness	199 (15.9)	7 (0.5)	31 (2.29)
Muscle pain	343 (27.4)	20 (1.5)	48 (3.54)
Palpitation	106 (8.5)	21 (1.5)	68 (5.02)
Fatigue	684 (54.7)	56 (4.1)	237 (17.49)

LC, long COVID; PCS, post COVID-19 syndrome.

### Longitudinal trends in prevalence of top reported long COVID symptoms

**Figure 2:**
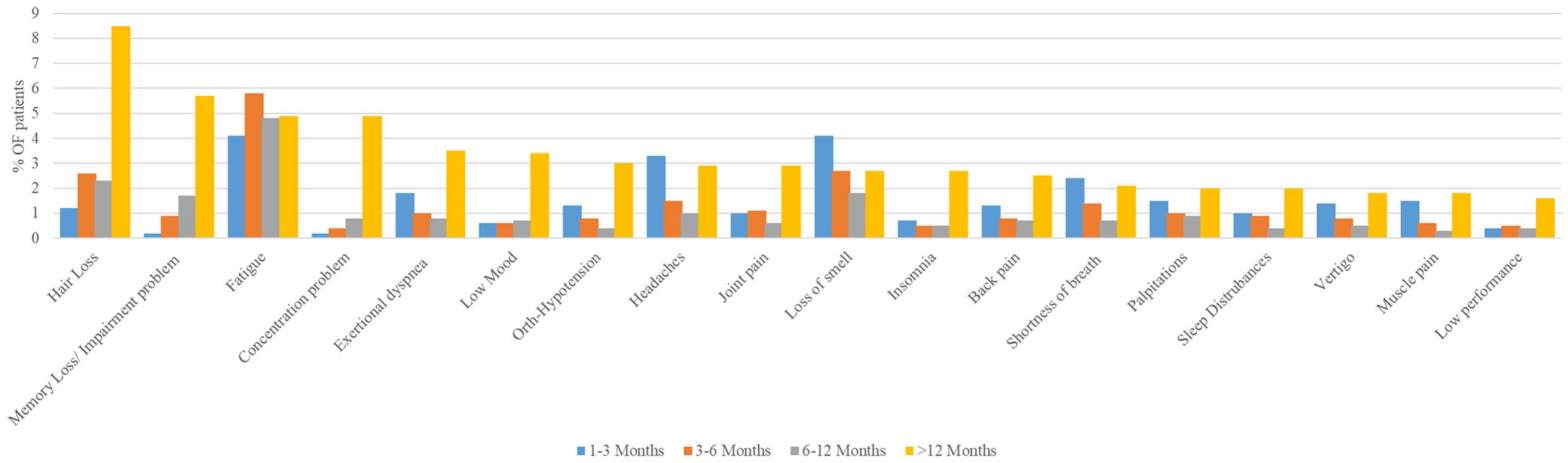
Longitudinal trends in prevalence of top reported long COVID symptoms among study participants across different time periods (*n* = 1,355).

Analysis of the top-reported symptoms among COVID-19 patients over different time periods (1–3 months, 3–6 months, 6–12 months, and > 12 months) revealed distinct trends (Figure 2). Symptoms such as hair loss, memory loss/impairment problems, concentration problems, low mood, joint pain, insomnia, and low performance increased over time, peaking at more than 12 months post-infection. Conversely, the prevalence of smell loss decreased after its initial peaks at 1–3 months. Several symptoms, including headache, shortness of breath, palpitations, vertigo, and muscle pain, exhibited a U-shaped trend, with initial peaks in the early months (1–3 months), a decrease at 3–6 and 6–12 months, and a subsequent increase at >12 months. Fatigue showed a relatively consistently high prevalence over time with a slight increase during the 3–6-month period but no significant long-term increase or decrease. Other symptoms, such as exertional dyspnea, orthostatic hypotension, back pain, and sleep disturbances, declined steadily after their initial peaks of 1–3 months, before re-emerging in prevalence after 12 months. These findings suggest a diverse range of symptom trajectories, some indicating long-term persistence, others resolving over time, and others showing fluctuating patterns.

### GEE multivariable gamma regression analysis results of risk factors for long COVID symptom count

Notably, women had a significantly higher mean LC symptom rate (17.4% more) compared to men (*p* < 0.001). However, the patients’ age did not converge significantly on their symptom rate across time (*p* = 0.228). Moreover, the patients’ measured count of symptoms was significantly higher (42.5% more) after 12 months on average compared to their baseline (<3 months) (p < 0.001), but the patients’ mean measured symptom rate at 6–12 months and during 3–6 months may not differ significantly compared to the 1–3 months ongoing symptomatic COVID-19 time (*p* > 0.050) (Table 4).

**Table 4:** GEE multivariable gamma regression analysis results of risk factors for long COVID symptom count among study participants (*n* = 1,355).

Variable	Adjusted risk rate (RR)	95% CI	*p*-value
Age (years)	1.00	(1, 1)	0.23
Gender			
Male	Reference		
Female	1.17	(1.1, 1.25)	<0.001*
Socioeconomic status			
Low–very low	Reference		
Medium–high	1.05	(1.01, 1.1)	0.022*
Education			
High school or less	Reference		
Diploma	1.01	(0.86, 1.18)	0.95
University degree	1.03	(0.94, 1.13)	0.51
Higher studies	0.67	(0.57, 0.8)	<0.001*
Body mass index (BMI)	1.01	(1, 1.02)	0.004*
COVID-19 vaccination status	0.96	(0.85, 1.09)	0.57
Number of acute COVID-19 symptoms	1.04	(1.03, 1.05)	<0.001*
Time			
Baseline <3 month	Reference		
3–6 months	0.96	(0.86, 1.07)	0.43
6–12 months	0.94	(0.83, 1.07)	0.37
>12 months	1.43	(1.28, 1.59)	<0.001*
(Intercept)	1.07	(0.62, 1.83)	0.82

*p < 0.05 considered statistically significant; CI, confidence interval.

Interestingly, the patients’ mean BMI score was positively associated with the mean number of LC symptoms. For each additional unit in the patients’ BMI, the mean predicted symptom rate tended to increase by a factor of 1.1% on average (*p* = 0.004), and heavier people reported a greater number of symptoms in general. The patients’ COVID-19 vaccination status had no significant influence on the mean number of reported persistent COVID-19 symptoms, (*p* = 0.568). In significant ways, the number of acute COVID-19 symptoms was positively associated with the mean number of LC symptoms (*p* < 0.001). For each additional symptom in the acute phase, the mean number of LC symptoms increased by an average of 4% (Table 4). Lastly, patients with higher education had a 33% lower risk of developing LC symptoms than those with a high school degree or lower (*p* < 0.001).

## Discussion

When looking at the results of our analysis of the responses of the 1,355 participants, it is evident that women had considerable odds of having a higher count of persistent symptomatology from their acute COVID-19 infection. Every unit increase in BMI in our study increased the risk of having higher symptoms count by 1%. Regarding economic capacity and education, there were elevated chances of persistent symptoms, only with an increase in the former (5.4%). Moreover, after looking at a timeline trend, it appeared that the number of LC symptoms was on average higher 12 months than before, and the number of acute COVID-19 symptoms was directly correlated with the number of lingering symptoms.

Research has demonstrated a 26% higher relative risk for individuals with COVID-19 to develop at least one of the LC symptoms. Several factors have been identified as contributing to this increased risk, including female gender, low socio-economic status, smoking, high BMI, and comorbidities (25). Among individuals with a proven history of COVID-19 infection, many risk factors were linked to the reporting of symptoms ≥12 weeks post-infection. Previous studies have consistently shown that women are more susceptible to experiencing long-lasting symptoms (26). In our analysis, the multivariate regression model revealed that women had a 17% chance (RR = 1.174) of having a higher count of persistent symptoms. This phenomenon is consistent with the findings of previous studies. While the literature offers many hypotheses on the underlying mechanisms that explain why women are at a higher risk of LC, among the most cited are immunological variations, such as reduced pro-inflammatory interleukin-6 (IL-6) production following viral infection in women, which would explain their more lasting symptoms (27). Additional variables, such as heightened psychological stress, isolation effects, and inactivity, may have also contributed to their higher risk (28).

Regarding BMI, larger target population studies found that a higher BMI is associated with more persisting symptoms, especially >30 kg/m^2^ as there is around a 10% relative increase in comparison to those with a BMI between 18.5–25 kg/m^2^ (28). Another study labeled BMI as the third strongest predictor of LC after increasing age and female sex (29). Notably, there was a positive correlation between the patients’ mean BMI score and the average number of LC symptoms. Every one-unit increase in BMI tended to increase LC symptoms by a factor of 1.1% on average. This relationship was statistically significant (*p* = 0.004). Age was not found to be a significant predictor in our study. However, the majority of other studies, including a 2023 meta-analysis of over 40 studies, suggested that older age was a significant contributing factor to LC (30). According to another study, this issue is primarily a liability for people who are already frail when infected (31).

Most research examining health disparities has utilized singular outlooks, focusing on individual factors such as sex, race, or deprivation, without adequately exploring the combined impact of intersecting inequalities on population health (32). For example, a study conducted in Brazil highlighted how the cumulative effects of poor health coverage, community disengagement, and low-income households are determinants that may play a significant role in the burden of COVID-19 disease and its complications (33). Their increased vulnerability to the virus may be linked to weakened immune systems owing to relatively higher stress levels (34). To complement this, a recent study inferred that individuals belonging to the most socioeconomically disadvantaged populations face the greatest susceptibility to LC, with an 11% higher risk than thriving individuals, and this disparity persists regardless of variations in the risk of initial infection (34). Our results however, showed that the scales minutely tip in favor of higher socioeconomic status correlated with persistent symptoms. However, this could be explained by the nature of our study population, as more than 90% had medium to high socioeconomic status. In the aforementioned study conducted in Brazil, researchers examined regions with comparatively broad healthcare coverage and observed an increase in the likelihood of identifying new cases only because their symptoms were more reportable and had better accessibility to healthcare facilities, which is also known as a detection bias (33). This was also the main takeaway message from a 2022 Swiss study that found that public health surveillance that determines epidemic severity depending on the number of positive testing cases alone was rather precarious, as it was highly limited to the availability of testing methods at certain locations (35). A higher education level was found to be a protective factor against LC in our data. This was consistent with a Spanish study that found that individuals with tertiary education were not only less likely to be affected by LC but also recovered faster if affected (36).

The regression table illustrates that the measured number of symptoms was significantly higher (42.5%) for >12 months than for the baseline phase (< 3 months). Correspondingly, this study shows how French patients’ COVID-related health conditions started to intensify 6 months after onset (37). Another study from South America showed that approximately 64% patients had at least one symptom reported 12 months after infection. The main risk factor is the mean number of symptoms observed during the acute phase (38). In our data, it was found that every symptom increases in acute presentation raised the risk of more persistent symptoms by approximately 4%. This is not surprising, as we know that the number of acute-phase symptoms correlates with disease severity, which tends to significantly increase the occurrence odds of LC, according to another UK study (39). Figure 2 shows that among the 18 top reported symptoms, 14 were reported at >12 months more than in the acute/ongoing phase.

### Strengths and limitations

As for the strengths, the questionnaire used was conducted via face-to-face interviews rather than online, prompting more genuine responses and immediate clarification by volunteers if any question was slightly confusing for the participants. Second, the study assessed syndrome prevalence 2 years post-COVID, a research area that is understudied. Moreover, this patient pool was evident because of its large sample size. It is also notable that a significant subgroup of the patients only needed at-home management, which is interesting as much of the literature regarding the topic always tends to target hospitalized patients or outpatient visitors.

This study has its limitations. First, it was conducted approximately 18 months after the peak of COVID-19 infections in the KSA (according to the WHO), so its retrospective nature may have led to a recall bias of acute and lingering symptoms. Second, the raw data were dependent on face-to-face interviews using questionnaires in public places, potentially leading to selection bias where individuals with LC symptoms may have been more motivated to participate; however, individuals with more severe symptoms may not have been equally represented due to difficulty in participating. Furthermore, the use of convenience sampling may limit the generalizability of the results to the broader population. The symptom ratings in the questionnaire could introduce a degree of subjectivity, and the lack of a control group consisting of non-COVID individuals complicates comparison. However, given the nature of a pandemic, it is challenging, if not impossible, to find individuals who have not been infected to act as a control group, which presents a methodological challenge. Additionally, the absence of objective clinical measures or biomarkers reduces the accuracy and precision of symptom assessment.

Due to these limitations, this study might not accurately reflect the experiences of the entire LC population. Our sample exhibited a significantly higher level of immunity and vaccination compared to other locations, where the majority of individuals may have received only one or two doses, or even none. This factor could possibly explain the distinct findings in our sample and may further limit the generalizability of the results to less vaccinated populations.

We recommend that the data presented be interpreted within the parameters of this study, and caution should be taken when generalizing the findings to all individuals with the condition.

## Conclusion

Potential factors linked to a higher number of LC manifestations included female sex, lower socioeconomic status, higher BMI, timing >12 months since COVID-19 infection, and a higher number of acute COVID-19 symptoms. Conversely, higher education offers a greater likelihood of protection against lingering symptoms. Thus, prospective health policy recommendations should integrate several elements of inequality, including sex, occupation, education, and socioeconomic disadvantages, when addressing the approach to and management of LC.

## Data Availability

The raw data supporting the conclusions of this article will be made available by the authors, without undue reservation.
