# Supplementary material for: Understanding long COVID: prevalence, characteristics, and risk factors in the Eastern Province of Saudi Arabia
Source: Front Med (Lausanne). 2024 Oct 4;11:1459583. doi: 10.3389/fmed.2024.1459583 (PMC11486694; doi:10.3389/fmed.2024.1459583)
Supplement: Supplementary file 1 [file Data_Sheet_1.PDF]

system	Manifestation
**Cardiovascular**	Chest pain<br>Palpitation<br>High blood pressure<br>Fainting<br>Thrombosis
**Pulmonary (Naso-oropharyngeal)**	Shortness of breath at rest<br>Shortness of breath with exertion<br>Anosmia<br>Dysphagia<br>Sore throat<br>Cough<br>Voice changes<br>Aphonia<br>Rhinorrhea<br>Sneezing<br>Sinusitis<br>Ear pain<br>Hearing loss<br>Tinnitus
**Gastrointestinal**	Diarrhea<br>Nausea<br>vomiting<br>Loss of appetite<br>Abdominal pain/ dyspepsia<br>Weight loss<br>Gastritis<br>Irritable bowel syndrome
**Reproductive**	Female:<br>Menorrhagia<br>Dysmenorrhea<br>Irregular/infrequent menstrual cycle<br>Infertility<br>Male:<br>Spermatogenic impairment<br>Decreased libido<br>Erectile dysfunction
**Neuro-Psychological**	Amnesia<br>Brain fog/ difficult thinking/ inability to concentrate<br>Neuropathy/neuralgia<br>Paresthesia/ tingling/ numbness<br>Tremors<br>Seizures<br>Headache<br>Vertigo/dizziness

	Mood change/ depression/ anxiety<br>Post traumatic stress disorder PTSD<br>Insomnia and sleep disorders<br>Visual disturbances<br>Language fluency difficulties
**Musculoskeletal**	Arthralgia<br>Myalgia<br>Weakness<br>Decreased skeletal mass
**Cutaneous**	Pruritus<br>Urticarial rash<br>Confluent erythematous/maculopapular/morbilliform rash<br>Papulovesicular exanthem<br>Livedo reticularis
**Other**	Fever /chills<br>Fatigue<br>Significant hair loss<br>Red eye / irritation<br>Unspecified pain<br>Hot flashes<br>Excessive sweating

Supplementary Materials Table S1. Most Common Long COVID Symptoms by Affected Body System (1–3).

Reference:

1. Aiyegbusi OL, Hughes SE, Turner G, Rivera SC, McMullan C, Chandan JS, et al. Symptoms, complications and management of long COVID: a review. J R Soc Med. 2021 Sep 15;114(9):428–42.

2. Li J, Zhou Y, Ma J, Zhang Q, Shao J, Liang S, et al. The long-term health outcomes, pathophysiological mechanisms and multidisciplinary management of long COVID. Signal Transduct Target Ther. 2023 Nov 1;8(1):416.

3. Calcaterra V, Zanelli S, Foppiani A, Verduci E, Benatti B, Bollina R, et al. Long COVID in Children, Adults, and Vulnerable Populations: A Comprehensive Overview for an Integrated Approach. Diseases. 2024 May 6;12(5):95.

supplementary materials Document S1. Research questionnaire

Dear Participant,

You are invited to participate in a research study aimed at understanding the prevalence, characteristics, and risk factors associated with Long COVID in the Eastern Province of Saudi Arabia. Your participation will provide valuable insights into this important public health issue.

Purpose of the Study
The purpose of this survey is to gather information on individuals who have had COVID-19 and their experiences with long-term symptoms, also known as Long COVID. Your responses will help us better understand the long-term impact of COVID-19, identify common symptoms, and analyze potential risk factors that may influence recovery.

Participation
Participation in this study is voluntary. The survey will take approximately 10 - 15 mins to complete. You may withdraw from the study at any time without any consequences, and you are free to skip any questions that make you uncomfortable.

Privacy and Confidentiality
Your privacy is extremely important to us. All responses will be anonymous and confidential. No identifying information (such as your name, email, or contact details) will be collected or linked to your answers. The data gathered will be used solely for research purposes and presented in a way that ensures participants cannot be identified. The results of this study may be published in academic journals or presented at conferences, but your identity will never be disclosed.

Consent
By proceeding with the survey, you are giving your informed consent to participate in this study. You acknowledge that:

- You have read and understood the information provided.
- You are at least 18 years old.
- You voluntarily agree to participate.

Thank you for your time and contribution to this important research. If you have any questions or concerns about the study, please contact Dr. Adam Aldhawyan, aaaldhawyan@iau.edu.sa.

1- Do you agree to participate?

- ○ Yes
- ○ No

2- Have you ever been diagnosed with COVID-19 infection (confirmed by serology)?

- Yes
- No

3- Age (years):

……………………………………

4- Sex:

- Male
- Female

5- Level of education:

- No formal education
- Primary school
- Secondary school
- Tertiary school
- Diploma
- Bachelor degree
- Master degree
- Doctoral degree

6- Nationality:

- Saudi
- Non-Saudi

7- Marital status:

- Single or never married
- Married
- Divorced
- Widowed

8- Economic level:

- 1 (Most deprived)
- 2
- 3
- 4
- 5 (Least deprived)

9- Occupation:

- Employed\Worker
- Not working
- Retired
- Student

10- Hight (CM):

………………………………………

11- Weight (KG):

………………………………………

12- Smoking status:

- Former (in the past)
- Current
- Never

13- Type of tobacco (You can choose multiple answers):

- Cigarettes
- E-cigarettes
- Cigars
- Hookahs
- None

14- Alcohol use:

- Yes
- No

15- Illicit drug use:

- Yes
- No

16- COVID-19 vaccination status:

- Never
- 1 Dose
- 2 Dose
- 3 Dose
- 4 Dose

17- Chronic disease (You can choose multiple answers):

- Diabetes mellites
- Hypertension
- Dyslipidemia
- Chronic kidney disease
- COPD
- Bronchial asthma
- Pulmonary fibrotic disease
- Immunodeficiency
- Rheumatological disease
- Malignancy
- Allergic disease
- Obesity
- Hepatic disease
- Ischemic heart disease
- Heart failure
- Arrhythmia
- Thromboembolism
- Cerebrovascular disease
- Neurological disease
- Migraine
- Obstructive sleep apnea
- Inflammatory bowel disease
- Hypothyroidism
- Hyperthyroidism
- Depression
- Anxiety
- Psychiatric disorder
- Sickle cell disease
- G6PD
- None

18- How many times you got COVID-19 infection (confirmed by PCR):

……………………………………

19- Presented with pneumonia:

- Yes
- No

20- Need for health care:

- Asymptomatic/No need
- Home management
- ER/out-patient services
- Hospital admission

21- Need for hospital admission:

- No need
- Word
- ICU

22- If yes, what is the duration of admission (days):

……………………………………

23- Need for oxygen supplementation:

- No need
- Nasal cannula
- Simple face mask
- Non-rebreather mask
- High-flow nasal cannula
- Intubation/Mechanical ventilation

24- Symptoms of acute presentation (You can choose multiple answers):

- Cough
- Expectoration
- SOB
- Chest pain
- Nasal congestion
- Runny nose
- Sinusitis
- Sore throat
- Fever
- Chills
- Back pain
- Joint pain
- Red eye
- Hypoxia
- Loss of taste (ageusia)
- Impaired taste (dysgeusia)
- Loss of smell (anosmia)
- Ear pain
- Hearing problem
- Visual problem
- Headache
- Paresthesia (tingling/numbness)
- Sleep disturbance

- Excessive sleepiness
- dizziness/drowsiness
- Sneezing
- Muscle pain
- Lack of appetite
- Nausea
- Vomiting
- Diarrhea
- Constipation
- Abdominal pain
- Palpitation
- Venous thrombosis
- fatigue
- Asymptomatic

25- Do you have any of these symptoms that persist or/ started after 4 weeks of confirmed COVID infection, and didn't explain by any alternative diagnosis, if yeas specify the duration?

- Cough
  - Yes

  - <3
  - 3-6
  - 6-12
  - >12
- Expectoration
  - Yes

  - <3
  - 3-6
  - 6-12
  - >12
- Shortness of breath
  - Yes

  - <3
  - 3-6
  - 6-12
  - >12
- Exertion dyspnea
  - Yes

  - <3
  - 3-6
  - 6-12
  - >12
- Chest pain
  - Yes

  - <3
  - 3-6
  - 6-12
  - >12
- Sinusitis
  - Yes

  - <3
  - 3-6
  - 6-12
  - >12

- Nasal congestion
  - Yes

- <3
- 3-6
- 6-12
- >12

- Pulmonary fibrotic change
  - Yes

- <3
- 3-6
- 6-12
- >12

- Hypoxia
  - Yes

- <3
- 3-6
- 6-12
- >12

- Vertigo
  - Yes

- <3
- 3-6
- 6-12
- >12

- Orth-Hypotension
  - Yes

- <3
- 3-6
- 6-12
- >12

- Palpitation
  - Yes

- <3
- 3-6
- 6-12
- >12

- Venous thrombosis
  - Yes

- <3
- 3-6
- 6-12
- >12

- Acute coronary syndrome
  - Yes

- <3
- 3-6
- 6-12
- >12

- Hypertension
  - Yes

- <3
- 3-6
- 6-12
- >12

- Diabetes
  - Yes

- <3
- 3-6
- 6-12
- >12

- Dyslipidemia
  - Yes

- <3
- 3-6
- 6-12
- >12

- Heart failure
  - Yes

- <3
- 3-6
- 6-12
- >12

- Loss of taste
  - Yes

- <3
- 3-6
- 6-12
- >12

- Loss of smell
  - Yes

- <3
- 3-6
- 6-12
- >12

- Impaired taste
  - Yes

- <3
- 3-6
- 6-12
- >12

- Hearing problem
  - Yes

- <3
- 3-6
- 6-12
- >12

- Visual problem
  - Yes

- <3
- 3-6
- 6-12
- >12

- Headache
  - Yes

- <3
- 3-6
- 6-12
- >12

- Anxiety
- Yes

- <3
- 3-6
- 6-12
- >12

- Depression
- Yes

- <3
- 3-6
- 6-12
- >12

- Post-traumatic stress
- Yes

- <3
- 3-6
- 6-12
- >12

- Brain fog
  - Yes
    - <3 ○ 3-6 ○ 6-12 ○ >12
- Memory problem
  - Yes
    - <3 ○ 3-6 ○ 6-12 ○ >12
- Slowdown thinking
  - Yes
    - <3 ○ 3-6 ○ 6-12 ○ >12
- Concentration problem
  - Yes
    - <3 ○ 3-6 ○ 6-12 ○ >12
- Paresthesia
  - Yes
    - <3 ○ 3-6 ○ 6-12 ○ >12
- Low mood
  - Yes
    - <3 ○ 3-6 ○ 6-12 ○ >12
- Fatigue
  - Yes
    - <3 ○ 3-6 ○ 6-12 ○ >12

❖ Hair loss

- Yes

  - <3 - 3-6 - 6-12 - >12

❖ Skin rash

- Yes

  - <3 - 3-6 - 6-12 - >12

❖ Joint pain

- Yes

  - <3 - 3-6 - 6-12 - >12

❖ Back pain

- Yes

  - <3 - 3-6 - 6-12 - >12

❖ Muscle pain

- Yes

  - <3 - 3-6 - 6-12 - >12

❖ Low performance

- Yes

  - <3 - 3-6 - 6-12 - >12

❖ Insomnia

- Yes

  - <3 - 3-6 - 6-12 - >12

❖ Sleep disturbance

- Yes

  - <3 ○ 3-6 ○ 6-12 ○ >12

❖ Excessive sleepiness

- Yes

  - <3 ○ 3-6 ○ 6-12 ○ >12

❖ Sexual dysfunction

- Yes

  - <3 ○ 3-6 ○ 6-12 ○ >12

❖ Dizziness/ drowsiness

- Yes

  - <3 ○ 3-6 ○ 6-12 ○ >12

❖ Fever

- Yes

  - <3 ○ 3-6 ○ 6-12 ○ >12

❖ Anemia

- Yes

  - <3 ○ 3-6 ○ 6-12 ○ >12

❖ Sweating

- Yes

  - <3 ○ 3-6 ○ 6-12 ○ >12

- ❖ Tinnitus
  - o Yes
    - o <3 o 3-6 o 6-12 o >12
- ❖ Kidney dysfunction
  - o Yes
    - o <3 o 3-6 o 6-12 o >12
- ❖ Dysuria
  - o Yes
    - o <3 o 3-6 o 6-12 o >12
- ❖ Hematuria
  - o Yes
    - o <3 o 3-6 o 6-12 o >12
- ❖ Oliguria
  - o Yes
    - o <3 o 3-6 o 6-12 o >12
- ❖ Menorrhagia
  - o Yes
    - o <3 o 3-6 o 6-12 o >12
- ❖ Oligomenorrhea
  - o Yes
    - o <3 o 3-6 o 6-12 o >12

- ❖ Polymenorrhea
- o Yes

  - o <3
  - o 3-6
  - o 6-12
  - o >12

- ❖ Hypothyroidism
- o Yes

  - o <3
  - o 3-6
  - o 6-12
  - o >12

- ❖ Hyperthyroidism
- o Yes

  - o <3
  - o 3-6
  - o 6-12
  - o >12

- ❖ No symptoms
- o Yes

Thank you for your participation
